# Effect of two milk supplements and two ways of administration on growth performance, welfare and fecal microbial ecology of suckling piglets

**DOI:** 10.3389/fvets.2023.1050414

**Published:** 2023-02-27

**Authors:** Federico Correa, Diana Luise, Clara Negrini, Roberta Ruggeri, Paolo Bosi, Paolo Trevisi

**Affiliations:** ^1^Department of Agricultural and Food Sciences (DISTAL), University of Bologna, Bologna, Italy; ^2^Agroscope, Pig Research Unit, Animal Production Systems and Animal Health, Posieux, Switzerland

**Keywords:** supplementary milk, gut microbiota, swine, piglet mortality, teat competition

## Abstract

**Introduction:**

The aim of this study was to evaluate the effect of two MS formulas, DanMilk™ (AB Neo, Denmark) (MS1) and Neopigg^®^ RescueMilk (Provimi, Netherlands) (MS2) administered manually and to compare two ways of administration (manual vs automatic) of MS1 on growth performance, health, fecal microbial profile, behavior, and skin lesions of piglets during suckling and post-weaning.

**Methods:**

Forty litters (528 piglets) were divided into 4 groups: 1) Control group receiving no MS (CON); 2) MS1 administered automatically (A-MS1); 3) MS1 administered manually (Ma-MS1) 4) MS2 administered manually (Ma-MS2). All groups had access to sow milk and creep feed. On day 5 after birth (d0), litters were equalized (13.2 piglets/litter ± 0.8 SD), thereafter no cross-fostering was allowed. Piglets were weighed at day 5 after birth (d0), at the end of milk supplementation (d14), at weaning (d21 of the trial, 26 days of age) and ten days post-weaning (d31). Piglet welfare was assessed using behavioral and lesion measures at d4 and d10. Feces were collected at d14 and d21.

**Results and discussion:**

During the suckling period, A-MS1 had lowest mortality (*p* < 0.05), while Ma-MS1 had lower mortality compared with CON and Ma-MS2 (*p* < 0.05). Negative social behavior at d4, was more frequent in MS groups (A-MS1, Ma-MS1, Ma-MS2) compared to CON group (*p* = 0.03). Growth performance and lesion prevalence were not affected by MS provision. During lactation, Ma-MS2 group had a higher percentage of piglets not eating during suckling at d18 compared with Ma-MS1 (*p* = 0.03). MS1 increased microbial diversity compared with CON at d14 (Chao1, *p* = 0.02; Shannon, *p* = 0.03) and compared with CON (Shannon, *p* < 0.05; InvSimpson, *p* = 0.01) and Ma-MS2 (Chao1, *p* < 0.05; Shannon, *p* = 0.05, InvSimpson *p* = 0.01) at d21. Groups that received MS1 were characterized by genera producing short-chain fatty acids (SCFAs), i.e., Lachnospiraceae (A-MS1) and Oscillospiraceae (Ma-MS1). MS composition and availability can contribute to reduce piglet's mortality during the suckling phase and can also affect intestinal microbiota by favoring the presence of SCFAs producing bacteria.

## 1. Introduction

In the last decade, the genetic progress aimed to increase the litter size, selecting the so-called “hyper-prolific” crossbreeds sows. The increased prolificacy has generated new challenges in managing sows and their litters (1). Given the limited ability of the sows to fully support the proper growth and development of a higher number of fetuses, large litters are characterized by high within-litter variation in birth weight and an increase of low-body-weight piglets with a lower vitality and thermoregulation issue. Furthermore, from a behavioral point of view, larger litters are found more likely to experience teat competition due to the limited access to functioning teats or insufficient milk provision by the sow. During the hours after birth, piglets usually identify and take ownership of a specific teat, or pair of teats. As lactation progresses, milk is released from the teats for a few seconds once or twice an hour (2). As a result, piglets compete for working teats, resulting in the creation of a stable “teat order” in which piglets occupy the same teat throughout each lactation event. This competition is inevitably exacerbated in larger litters (1), and can produce unevenness among littermates, increasing the risk of starvation, disease susceptibility and crushing by the sows (1), which in turn may cause productivity losses and increase the mortality rate (3). In addition, Ocepek et al. (4) observed that, on average, a piglet in a large litter spend more time stimulating the udders causing him to lose more energy, which may impair its vitality and survivability during suckling. This behavior is associated with the decrease in the functional teats per piglets ratio resulting in an increased teat competition and frustration for the piglets, which can represent a risk factor for their health, especially for the submissive ones (5). In addition, piglets rely heavily on sow milk for their growth and development, however, the amount of milk a piglet receives can be affected by factors such as the number of piglets nursing, the size and health of the piglets, and the stage of lactation (6). In addition, milk production can be compromised when sows experience thermal discomfort (7). Despite the lactational ability of the sow, it is not uncommon for the milk production to be not sufficient to sustain the optimal growth needs of all the piglets. This is particularly seen around 8–10 days of age and becomes more pronounced as lactation continues. Moreover, this problem was exacerbated in the last year with the use hyper-prolific genetic lines (8). A strategy, that becomes largely adopted in commercial herds to address this issue, is the use of milk supplements (MS) as a complement to the sow milk and creep feed, as it can sustain the full growth potential of the piglets. Indeed, as reviewed by Huting et al. (9), particularly during the first 2 weeks of lactation, around 51% of the litter consume MS compared with the 5% of litter that eat creep feed. Recent evidence suggests the positive effect of MS on the body weight (BW) at weaning and survivability of the large litters (17 piglets) (10). Nevertheless, the effect of MS on the performance of pigs is not fully demonstrated. Some evidence shows that MS can reduce the BW variation within the litter and pre-weaning mortality, but not the growth performance during suckling and post-weaning in low birth weight piglets (10, 11). These conflicting results can be linked to the composition of the MS as the protein content can affect the development of intestinal microbiota, barrier function and immune system (12), as well as to the way of administration. Moreover, there is some evidence that MS supplementation during the suckling period can favor the small intestinal growth and cell proliferation, modulating the intestinal microbiota by increasing its diversity, and improving the concentrations of short-chain fatty acids (SCFA) (13). Recently, industries have commercialized systems which can provide MS *via* an automatic dispenser placed inside the pen. The advantages of this system can be ascribed to the lower workload for the farmers but also to a reduced waste and reduced microbial contamination of the MS, which is provided fresh and in a small portion, when required, during the whole day. Therefore, authors hypothesize that both the composition and the method of distribution of the MS can modify the social behavior and the intestinal maturation of the piglets and therefore the productive performance and health during the lactation phase. Nonetheless, the impact of the different sources of MS and distribution systems is scarcely investigated (14).

The present study was designed to satisfy two aims: (1) to compare the effect of two MS formulas distributed in the same conditions (manual system MS1 and MS2); (2) to compare the effect of the same MS provided with a different distribution system (continuous availability supplied through an automatic system, or the restricted availability using milk cups filled twice a day), provided during the suckling period on litter performance, behavior, lesions incidence, and fecal microbial profile of suckling and weaned piglets.

## 2. Materials and methods

The present study involved 40 litters (528 piglets) raised on a commercial farm located in northern Italy. On day 5 after birth (day 0–d0), the litters were divided into 4 groups (10 litters/group) balanced per litter size (13.2 piglets/litter ± 0.8 SD), litter weight (1,934.2 ± 397.9 SD), dam's parity (3.4 ± 0.8 SD) and number of functional tests (12.3/litter ± 0.9 SD) (Table 1) to have a fractional design. The litters were assigned to one of the following groups: (1) Control group receiving no milk supplements (CON); (2) MS1 (DanMilk™, AB Neo, Denmark) administered automatically (A-MS1); (3) MS1 administered manually (Ma-MS1) (4) MS2 (Neopigg^®^ RescueMilk, Provimi B.V., Rotterdam, Netherlands) administered manually (Ma-MS2).

**Table 1:** Experimental design.

**Group^a^**	**N. litters**	**N. of total pigs**	**Parity number, median (range)**	**Litter size median (range)**	**Piglets body weight day 0 (day 5 after birth), g (sd)**
CON	10	133	3	13	1,841.9
			(2,7)	(12,14)	(488.8)
A-MS1	10	133	3	13	1,975.5
			(1,6)	(12,14)	(470.6)
Ma-MS1	10	132	4	13	1,965.0
			(1,5)	(12,14)	(354.2)
Ma-MS2	10	130	2	13	1,954.8
			(1.6)	(12,14)	(295.7)

^a^Descriptive statistics of sows' parity, litter size and piglets body weight. Group^1^: CON, control group without milk supplementation; A-MS1, DanMIlk Supreme (AB Neo a/s, Denmark), provided automatically; Ma-MS1, DanMIlk Supreme (AB Neo a/s, Denmark), provided manually twice a day; Ma-MS2, Neopigg RescueMilk (Provimi B. V., Netherlands), provided manually twice a day.

In the A-MS1 litters, the farrowing crates were equipped with an automatic drinking system (Pump'n'Grow Fresh system, AB Neo, Denmark), and MS1 was available at piglets by pressing the cup 12 h/day (from 7 a.m. to 7 p.m.). For MS2, it was not possible to administer it automatically since the milk distribution system present within the farm did not have two separate distribution lines. In Ma-MS1 and Ma-MS2 groups, MS was provided manually twice a day (around 7 a.m. and 4 p.m.) in a dish (28.5 cm diameter) attached to the slats at the rear of the farrowing crate, when leftover milk was present this was removed, and the cup was wash and refilled with fresh milk. The MS powder was reconstituted with water at room temperature according to the manufacturer's instructions. The composition of MS is reported in Table 2.

**Table 2:** Milk supplements composition.

**Analytical components**	**MS 1^a^**	**MS 2^b^**
Crude protein, % DM	20.0	20.9
Crude lipid, % DM	17.0	10.5
Crude ashes, % DM	5.7	9.5
Crude fiber, % DM	0.0	0.1
Lactose, % DM	40.0	-
Lysine, g/kg DM	16.0	17.5
Methionine, g/kg DM	4.5	5.0
Ca, g/kg DM	6.0	5.7
P, g/kg DM	4.5	7.9
Na, g/kg DM	3.4	7.8
ME, kcal	3,685	3,941

Analytic composition of the MS used in the trial MS1 (DanMIlk Supreme, AB Neo a/s, Denmark) and MS2 (Neopigg RescueMilk, Provimi B. V., Netherlands).

^a^**Nutritional additivies** (per kg): A-vitamin, E672, IE = 25,500; D3-vitamin, E671, IE = 2,550, DL-Alpha-tocopherol, mg = 130, Zinc (Zn), mg = 100, Copper (Cu), mg = 130, lodine (1), mg = 2.0; Fe, mg = 150. **Zootechnical additives**: Bacillus subtilis 0.6 Mia CFU, Bacillus licheniformis 0.6 Mia CFU.

^b^**Nutritional additives** (per kg): 3a672a Vitamin A= 24,999 IU; E671 Vitamin D3 =5,000 IU; 3a700 Vitamin E = 150 IU; Monohydrat/El Iron (Ferrous sulfate, monohydrate) = 87 mg; Pentahydrat/E4 Copper (Cupric sulfate, pentahydrate) = 150 mg. Zinc (Zinc oxide) = 50 mg. Manganese (Manganous sulfate, monohydrate) = 100 mg. 36,202 Iodine (Calcium iodate, anhydrate) = 2.0 mg, E8 Selenium (Sodium selenite) = 0.25 mg. **Zootechnical additives** (per kg): 4b1700i Bacillus licheniformis (DSM5749) and Bacillus subtilus (DSM5750) 1:1 1.3 E+09 CFU. **Sensory additives** (per kg): E954 Sodium saccharin 112 mg.

The MS were available from d0 (day 5 after birth) to d14 of the trial, thereafter only creep feed was provided until weaning (d21 of the study, 26±2 days of age) in a dish (28.5 cm diameter) attached to the slats at the back of the farrowing crate. During the trial, two phases of a dry mashed creep feed were provided for all the litters: Phase 1 = from d0 to d10 of the trial, consisted of corn, wheat feed flour, soybean protein and whey powder; Phase 2 = from d10 to d21 of the trial, consisted of barley flakes, wheat, whey powder, food industry by-products, plasma and was medicated with ZnO 3,000 mg. Calculated chemical composition of creep feed is reported in Table 3. Creep feed was provided through a feeder (28.5 cm diameter) attached to the slat.

**Table 3:** Creep feed composition.

**Calculated chemical composition (%)**	**Phase 1**	**Phase 2**
Crude protein	18.00	17.50
Crude lipids	8.60	7.50
Crude fiber	1.00	2.50
Crude ashes	4.10	5.00
Ca	0.50	0.52
P	0.48	0.62
Na	0.31	0.37
Lysine	1.20	1.41
Methionine	0.41	0.39
Gross energy (Kcal/kg)	4,102	4,026

Sows included in the study (DanBred^®^) were raised in conventional farrowing crates of 4.5 m^2^ (2.5 x 1.8 m), that where equipped with wood blocks and paper strings. The trial was performed from June to August 2020; the average temperature in the farrowing crate during the entire period was 26.71°C (± 1.59°C). The creep area was not covered and was equipped with a heating lamp, that was turned on 24 h/day. The temperature was on average 27.32°C (±1.74°C) in the creep area. Lights were turned on between 7 a.m. and 16 p.m. The health status of the animals was daily controlled. Treated or non-eating sows were removed from the trial. Three days after birth, piglets underwent tail docking using cauterizing pliers and male piglets were castrated.

At weaning (d21 of the trial, 26 ± 2 days of age), piglets were randomly regrouped in 22 pens of about 30 (8 x 2 m) equipped with sub-optimal enrichment materials (iron chain coupled with wood blocks), following the common management used in the farm. Piglets were fed the same phase 2 creep feed diet until the end of the trial (d10 post-weaning).

### 2.1. Litter performance

Piglet's mortality and health were monitored daily until the end of the trial. Piglets were excluded from the trial in case of severe impairment of the health conditions characterized by limited movements and attention, no milk intake and visible loss or arrest of growth. Piglets were individually weighted at the beginning of the trial (d0, 5 days after birth), at the end of milk supplementation (d14 of the trial), at weaning (d21 of the trial) and 10 days post-weaning (d31 of the trial).

### 2.2. Behavioral and lesion assessment

Welfare of piglets was assessed using behavioral and lesion measures. Selected welfare indicators were assessed on a subset of litters (5 litters/group). Litters were chosen to be balanced for initial litter-size, litter weight and dam's parity. Observations were made at d4 of the trial, and at d10 of the trial (before the milk supplementation was interrupted) and at d18 of the trial (before weaning), when the milk supplementations were interrupted to evaluate if this practice can increase the negative social interactions linked to teat competition.

To assess behavior, the collected parameters were divided into behavioral measures (BMs) and behavior during lactation (LBs). The procedure followed the protocol described by Vitali et al. (5) and was carried out by trained observers.

Briefly, BMs included category of behavior as described in the Welfare Quality protocol (15). The category of behavior included “social behavior,” “exploratory behavior,” “other active behaviors,” and “inactive behavior and are detailed in Table 4. The frequency of “social,” “exploratory,” and “other active behaviors” was determined on the total of active behavior in each litter. Frequency of “inactive behavior” was calculated on the total behavior observed, as explained in the Welfare Quality protocol for pigs (15). Observed individual behavior included negative social behavior (such as fighting and ear, tail and body biting); and positive social behavior (play). The behavior assessment was evaluated outside the pen, between 9:00 and 11:00, by direct observations of all the piglets in each litter. Piglets where individually identified when possible through a numbered ear tag that was applied at the start of the trial. For each litter three observations of 6.5 min were performed with an interval of 5 min between each observation. For each behavior category, the average of the three observations was calculated. They were calculated as the percentage of the animals exhibiting the behavior over the total of animals in the litter: [(n. of piglets demonstrating the behavior/total of piglets in the litter) ^*^100].

**Table 4:** Animal-based measures.

**Type**	**Measure**	**Description**
BM	Negative social behaviors	Negative social behavior included any aggressive social behavior or biting causing a response from the disturbed animal.
BM	Positive social behaviors	Positive social behavior consisted of sniffing, licking, play and moving gently away from the other animal without aggressive or fight reaction from this individual.
BM	Explorative behaviors (pen and environmental enrichment–directed)	Explorative behaviors pen-directed is defined as sniffing, nosing, licking or chewing any features within the pen. Exploring enrichment material is defined as play/investigation toward straw or other enrichment material.
BM	Other active behaviors	Any active behaviors not included in the previous categories, such as eating, drinking or air sniffing.
BM	Inactive behaviors	Any behaviors when the animal remains motionless thus without any activities.
LB	Teat competition	Piglets that are fighting for the same teat. Fights were observed both in the upper and lower teat row when possible.
LB	Not eating	Piglets who did not eat from the sow neither tried to access to supplementary milk.
LB	Supplementary milk	Piglets that, during the nursery event, consumed the supplementary milk instead maternal one.
LM	Skin lesions	Considers the 5 separate areas (ear, fronts, middle, hind-quarters, legs). Score was 0 = up to 4 lesions visible; 1 = 5–10 lesions visible; 2 = 11–15 lesions visible.
LM	Tail lesions	0 = absence of lesions; 1 = superficial biting along the length of the tail but no evidence of swelling or blood; 2 = fresh blood visible on the tail, or presence of a scar, swelling, or a missing a part of the tail.

Description of the animal-based measures used to assess piglet' welfare through the trial. BM, Behavioral measure; LB, Lactation Behavior; LM, Lesion measurement.

LBs were recorded using the methods modified from Balzani et al. (16), Fraser (17) and Milligan et al. (18). The behaviors were classified as: (i) “Teat competition” when piglets are fighting for the same teat from the upper or lower row, (ii) “Not eating” when piglets did not eat from the sow or tried to access supplementary milk, (iii) “Supplementary milk” when piglets consumed the supplementary milk instead maternal one during the nursery event. The number of piglets involved in (i) teat competition, (ii) not eating or (iii) consuming the supplementary milk was recorded by direct observation of three suckling events per sow, then the data were transformed in percentage on the total of piglets in the litter. Moreover, the total number of teats, the number of non-functional teats and the level of teat exposition (referred the sow's propensity to expose the udder) during the lactation event were recorded for each sow. The level of teat exposition was classified according to Balzani et al. (16) as follows: 1 = sow exposed only the upper row of the teats; 2 = sow exposed three-quarters of the teats, 3 = sow exposed both teat rows.

Lesion measures (LMs) were assessed on ears, legs, tail, front, middle and forelimbs of each piglet. LMs were scored according to the method described in the Welfare Quality^®^ (2009). Skin lesions were scored from 0 to 2 as follows: 0 = up to 4 lesions, 1 = 5–10 lesions, 2 = more than 11 and tail lesions were scored from 0 to 2 as follows: 0 = absence of lesions; 1 = superficial biting along the length of the tail but no evidence of swelling or blood; 2 = fresh blood visible on the tail, or presence of a scar, swelling, or a missing a part of the tail. The prevalence of each score was calculated for each litter. Then a final lesion score index (LSI) was calculated in each area as follows (range 0 to 200): LSI = [prevalence of lesion with score 1 + (2^*^ prevalence of lesion with score 2)].

### 2.3. Fecal microbial analysis

At d14 and d21 of the trial, a total of 120 fecal samples (15 samples/group/time point) were collected from the same three piglets per litter chosen by a subset of 5 litters per group, balanced for initial litter-size, litter weight and dam's parity. In the selected litter feces were collected from subjects which had average body weight within the litter. The fecal samples were collected in a sterile tube, immediately frozen and then stored at −80°C for microbiological analysis. Total bacterial DNA was extracted from the fecal samples using FastDNATM Spin Kit for Soil (MP Biomedicals, Europe, LLC) following the manufacturer's instructions. The quantity and purity of the isolated DNA were checked by spectrophotometry on the NanoDrop (Fisher Scientific, 13 Schwerte, Germany), and agarose gel electrophoresis. Then, the characterization of the microbial profile was performed by sequencing the V3–V4 regions of the 16S rRNA gene. Briefly, amplicons were produced using the primers: Pro341F:5'-TCGTCGGCAGCGTCAGATGTGTATAAGAGACAGCCTACGG GNBGCASCAG-3, and Pro805R: 5'-GTCTCGTGGGCTCGGAGA TGTGTATGAGACAGGACTACNVGGGTATCTTCC-3' (19) and the PlatinumTM Taq DNA Polymerase High Fidelity (Termo Fisher Scientific, Italy). The libraries were prepared using the standard protocol for MiSeq Reagent Kit v3 and sequenced on MiSeq platform (Illumina Inc., San Diego, Ca, USA).

### 2.4. Statistical and bioinformatical analysis

#### 2.4.1. Litter performance

Pre-weaning mortality and exclusion of piglets were summed and analyzed using the litter as experimental unit and data were analyzed using a general linear model (GLM) applying a Poisson distribution and including the group, sow parity and day of birth as independent factors. A *post-hoc* Tukey's test was then performed to identify differences among groups. In order to answer the aforementioned aims, specific pairwise contrasts, including CON vs. MILK = Control group vs. litters receiving milk supplementation (thus A-MS1, Ma-MS1 and Ma-MS2–aim “i”), Ma-MS1 vs. Ma-MS2 (aim “ii”), Ma-MS1 vs. A-MS1 (aim “iii”), and Automatic vs. Manual Supplementation (A-MS1 vs. Ma-MS1+Ma-MS2 – aim “iv”), were carried out. In the post-weaning phase, mortality was calculated as the percentage of dead piglets per groups and differences were tested using a chi-squared test.

Data on pre-weaning growth performance were analyzed using the PROC MIXED of SAS software (v9.4) (SAS Institute Inc., Cary, NC, USA), including group as fixed factors, litter size and initial BW as covariates and the litter as the random factor. The interaction between BW and group was also investigated. The model of the post-weaning growth performance included the group as fixed factors, initial BW as covariates and the litter and box as random factors. Dam's parity, sex of piglets and date of birth were initially included in the model but then removed following a stepwise backward elimination approach. Data were analyzed considering the litter as the experimental unit during the suckling phase. While for the post-weaning phase the pig was used as experimental unit.

#### 2.4.2. Behavior and lesion assessment

Data on BMs (“Inactive behavior,” “Negative social behavior,” “Positive social behavior,” “Pen exploration,” “Environmental enrichment exploration” and “Other active behavior”) and LBs (“Teat competition,” “Not eating” and “Supplementary milk”) were analyzed considering the litter as the experimental unit. BMs were analyzed with an ANOVA model including group as independent factors and litter size and observer as a covariate. LBs were analyzed using an ANOVA model including the number of functioning teats and teat exposition as independent factors, and litter size and observer as a covariate. Indexes for skin and tail lesions were assessed using the litter as the experimental unit. Lesions were analyzed using an ANOVA model including group as fixed factor, initial BW as covariate and the litter as the random factor. The same specific pairwise contrasts were performed also for these series of parameters. The observer was initially included in the model but then removed following a stepwise backward elimination approach.

#### 2.4.3. Microbial bioinformatic analysis

Microbiota analysis was performed using DADA2 pipeline (v138.1) (20) and taxonomic categories were assigned using Silva Database (release 138) as reference (21). Alpha (Shannon, Chao1 and InvSimpson indices) and Beta diversity (calculated as Bray Curtis distance matrix at ASV level), as well as the abundance of taxonomic categories, were analyzed at d14 and d21 separately using R software 4.1, with phyloseq (v1.38) (22), vegan (v2.6) (23) and car (v3.1) (24) packages. Statistical analysis on alpha diversity indices was carried out with an ANOVA model, considering the sequencing depth and the group as fixed factors. A *post-hoc* Tukey's test was then performed to identify differences among groups. Beta diversity was analyzed with a PERMANOVA model (“Adonis” procedure) including the group, the time and their interaction with 100 permutations. Prior to the PERMANOVA, the homogeneity of dispersion between groups was tested using the *permdisp* function, with 100 permutations. Furthermore, in order to identify the discriminant taxa of each group, the multivariate sparse Partial Least Squares Discriminant Analysis (sPLS-DA) supervised approach, implemented in the mixOmic package (v6.18.1), was carried out on the data microbial at d14 and d21 separately (25). Microbial data, aggregated at genus level, were previously normalized using the total sum scaling normalization coupled with the centered log-ratio transformation.

Statistical analysis was conducted in the R environment (26) using car, lsmeans and lmer packages and on SAS software using the PROC MIXED method. For the growth performance, behavioral analysis, and alpha diversity the same specific pairwise contrasts were performed. *p*-value significance was set at *p* < 0.05 in all the analyses while values *p* < 0.10 were considered as a trend.

## 3. Results

### 3.1. Litter performance

The sows remained healthy during the overall trial and the litters did not show any abnormal health impairment.

Growth performances and piglet mortality during the suckling phase are reported in Table 5, the MS administration did not affect the growth performance of the piglets. Overall, piglets of A-MS1 group had an increased survivability compared with the CON, Ma-MS1 and Ma-MS2 groups both during the MS administration (d0-d14) (A-MS1 vs. CON, *p* < 0.001; A-MS1 vs. Ma-MS1, *p* < 0.05; A-MS1 vs. Ma-MS2, *p* < 0.001) and during the whole suckling period (d0-d21) (A-MS1 vs. CON, *p* < 0.001; A-MS1 vs. Ma-MS1, *p* = 0.01; A-MS1 vs. Ma-MS2, *p* < 0.001). The Ma-MS1 group had a lower mortality when compared with CON and Ma-MS2 (Ma-MS1 vs. CON, *p* = 0.02 in d0-d21 and *p* < 0.001 in d0-d14, Ma-MS1 vs. Ma-MS2, *p* < 0.001 in d0-d21 period and *p* < 0.001 in d0-d14). Conversely, no differences in mortality were observed after the MS suspension (d14-d21).

**Table 5:** Effect of milk supplementation on piglets' litter performance during the suckling phase.

**Item**	**Mean** ^**1**^				**SEM**	**Group, *p*-value^2^**
	**CON**, ***n*** = **10**	**A-MS1**, ***n*** = **10**	**Ma-MS1**, ***n*** = **10**	**Ma-MS2**, ***n*** = **10**		
**Body weight (g)**						
BW d0	1,946	1,991	2,014	1,897	140	0.94
BW d14	5,173	5,319	5,450	5,315	148	0.52
BW d21	6,586	6,496	6,884	6,711	217	0.61
**Average daily gain (g/day)**						
ADG d0-14	228	238	249	239	10	0.61
ADG d14-21	200	163	203	202	14	0.16
ADG d0-21	220	213	234	227	23	0.44
**Mortality (%)**						
d0–d21	9.21^c^	3.09^a^	6.71^b^	9.09^c^	1.98	< 0.001
d0–d14	8.38^c^	3.09^a^	6.00^b^	8.38^c^	1.88	< 0.001
d14–d21	0.90	0	0.77	0.77	0.62	N.S.

^a, b, c^ = values with different superscript differs (p < 0.05).

^1^CON, control group without milk supplementation; A-MS1, MS1, provided automatically; Ma-MS1, MS1, provided manually twice a day; Ma-MS2, MS2, provided manually twice a day.

^2^No significant effect for CON vs. MS, CON vs. litters receiving MS (A-MS1, Ma-MS1 and Ma-MS2), for MS formula (Ma-MS1 vs. Ma-MS2) and for Automatic vs. Manual Supplementation (A-MS1 vs. Ma-MS1+Ma-MS2).

Growth performance and mortality during the post-weaning phase are reported in Table 6. No significant differences among groups were observed for the growth performance and mortality (d21-d31).

**Table 6:** Effect of milk supplementation on piglets' performance 10 days post-weaning.

**Item**	**Mean** ^**a**^				**SEM**	***p*** **-value**	
	**CON**, ***n*** = **133**	**A-MS1**, ***n*** = **133**	**Ma-MS1**, ***n*** = **132**	**Ma-MS2**, ***n*** = **130**		**Group**	**Sex**
BW d31 (post-weaning), g	7,925	7,833	7,941	7,990	186	0.94	0.93
ADG d0-31, g/day	195	191	202	195	7	0.69	0.85
ADG d 21-31 (post-weaning), g/day	142	144	135	131	9	0.67	0.77
Mortality d21-31 (%)	1.65	1.55	3.25	0.85	0.51	0.29	-

^a^CON, control group without milk supplementation; A-MS1, MS1, provided automatically; Ma-MS1, MS1, provided manually twice a day; Ma-MS2, MS2, provided manually twice a day. (The analysis was performed using the piglets as experimental unit).

### 3.2. Behavioral measures

All the results of the behavioral measures (BMs) are reported in Supplementary Table 1. Behavior analysis showed that pen exploration at d4 was more frequent in the Ma-MS2 group than in the CON group (19.85 vs. 3.28%, *p* = 0.02). In addition, at d4, the CON group showed a trend for more inactive behaviors (*p* = 0.06) as well as a significantly lower negative social behavior (*p* = 0.03) and lower pen exploration (*p* = 0.02) than the MS groups. No differences between groups were observed for the other BMs.

**Figure 1:**
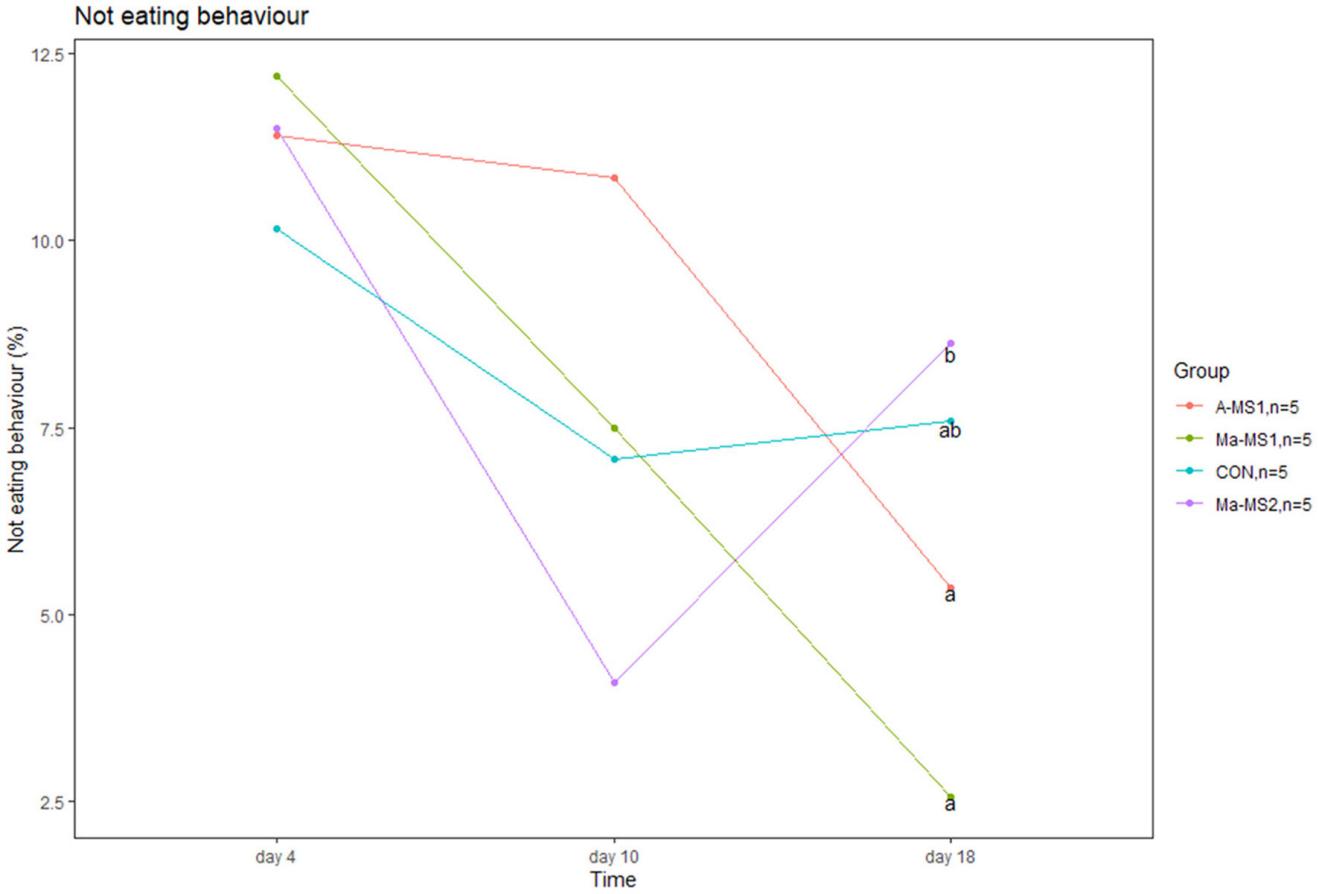
Line plot showing the percentage of piglets not eating during the suckling event at day 4, 10 and 18 of the trial. ^a, b^values with different superscript differs (*p* < 0.05).

Results of the lactation behaviors (LBs) are reported in Supplementary Table 2. Teat competition did not differ between groups during suckling. Piglets from the Ma-MS2 group tended to show a higher percentage of teat competition at d10 compared with piglets from the Ma-MS1 group (*p* = 0.05). In addition, Ma-MS2 group had a higher percentage of piglets not eating during the suckling at d18 compared with A-MS1 and Ma-MS1 groups (*p* = 0.01 and *p* = 0.03, respectively) (Figure 1). No effects were observed for the other time points and parameters.

### 3.3. Lesion measurement

Results on the lesion measurement (LMs) are reported in Supplementary Table 3. At d4, piglets from the A-MS1 group had higher LSI in the ear (*p* = 0.03) and tended to have a higher LSI in the middle area (*p* = 0.05) compared with the piglets in the groups receiving the manual milk supplementation. At the other days of observation, the milk supplementation did not show significant effect except for the lesion scores on the hind quarter at d10 (*p* = 0.03), which were higher in the Ma-MS1 group compared with the Ma-MS2 group (*p* = 0.02).

### 3.4. Fecal microbiota

Four samples were excluded due to the low number of reads (lower than 5,000). After quality check and filtering, a total of 6,177,332 raw reads were attributed to a total of 3,062 amplicon sequence variants (ASVs) distributed among samples as shown in Supplementary material 1. The relative rarefaction curves (Supplementary Figure 1) showed a tendency to plateau for all samples, suggesting that the sequencing depth was sufficient to describe the variability within the analyzed microbial communities.

**Figure 2:**
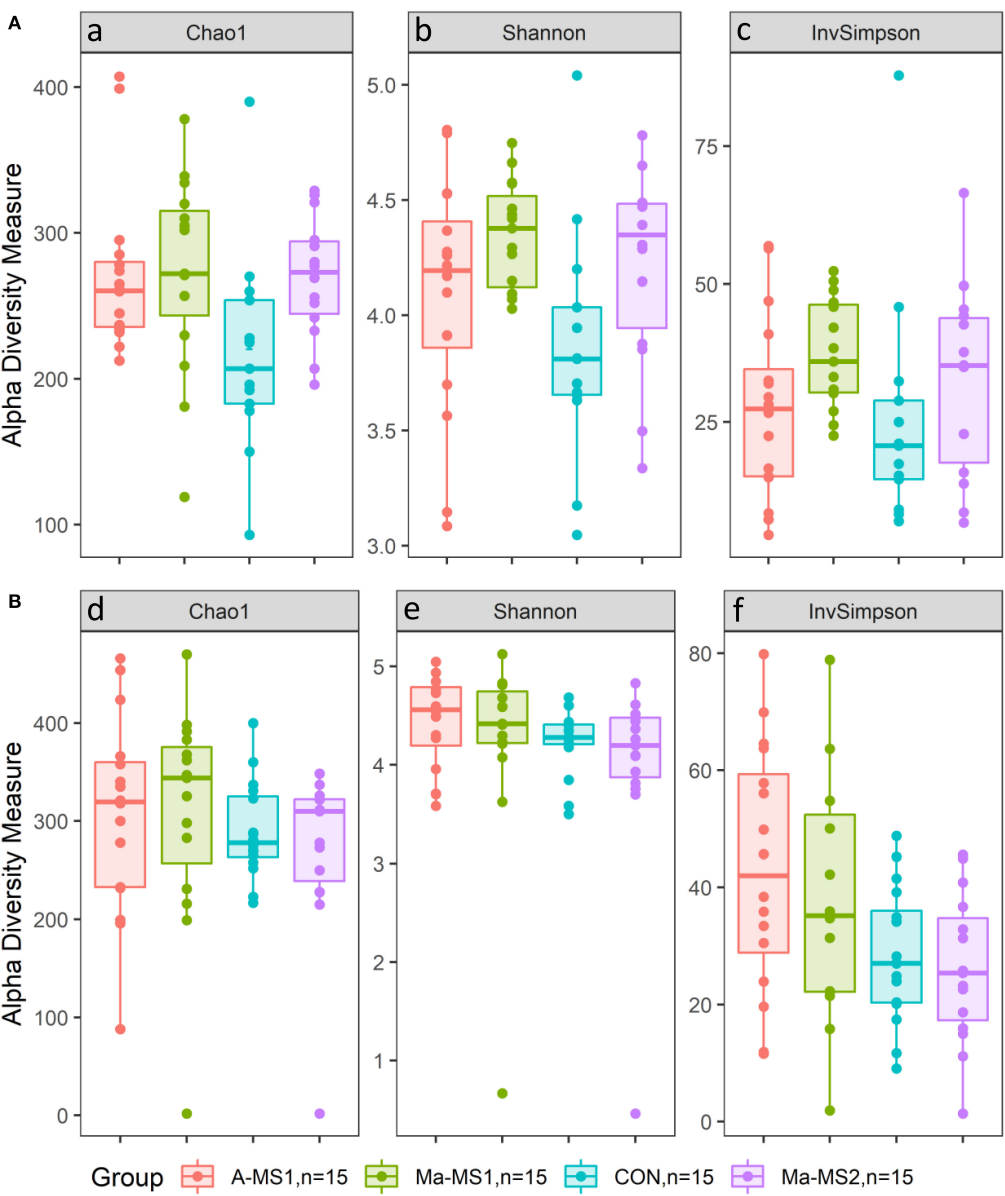
Box plot of alpha diversity indices values according to the group. **(A)** d14; **(B)** d21; CON = control group without milk supplementation; A-MS1 = MS1, provided automatically; Ma-MS1 = MS1, provided manually twice a day; Ma-MS2 = MS2, provided manually twice a day. ^a^CON vs. A-MS1, *p* = 0.05; CON vs. Ma-MS1, *p* = 0.02; CON vs. Ma-MS2, *p* = 0.09; CON vs. MS, *p* = 0.02; CON vs. A-MS1+Ma-MS1, *p* = 0.02. ^b^CON vs. Ma-MS1, *p* = 0.01; CON vs. Ma-MS2, *p* = 0.07; CON vs. MS, *p* = 0.03; CON vs. A-MS1+Ma-MS1, *p* = 0.03. ^c^CON vs. Ma-MS1, *p* = 0.10. ^d^CON vs. A-MS1, *p* = 0.07; CON vs. A-MS1+Ma-MS1, *p* = 0.06; Ma-MS2 vs. A-MS1+Ma-MS1, *p* = 0.05. ^e^CON vs. A-MS1, *p* = 0.08; CON vs. Ma-MS1, *p* = 0.09; CON vs. A-MS1+Ma-MS1, *p* < 0.05; Ma-MS2 vs. A-MS1+Ma-MS1, *p* = 0.053. ^f^CON vs. A-MS1, *p* = 0.01; CON vs. Ma-MS1, *p* = 0.06; CON vs. MS, *p* = 0.07; CON vs. A-MS1+Ma-MS1, *p* = 0.01; Ma-MS2 vs. A-MS1+Ma-MS1, *p* = 0.01; Automatic vs. Manual, *p* = 0.01.

For the alpha diversity measures, at d14, the Chao1 and the Shannon indices were significantly higher in the groups receiving MS (A-MS1, Ma-MS1 and Ma-MS2) compared with the CON group (*p* = 0.02 and *p* = 0.03). In addition, the Chao1 index was higher in the A-MS1 and Ma-MS1 groups compared with the CON group (*p* = 0.05 and *p* = 0.02, respectively) and tended to be higher in the Ma-MS2 group compared with the CON group (*p* = 0.09). Shannon index was higher in the Ma-MS1 group compared with the CON (*p* = 0.01) and tended to be higher in the Ma-MS2 group (*p* = 0.07) compared with the CON, while InvSimpson index was not influenced. Considering the contrasts performed at d21, the CON and the Ma-MS2 groups showed the lowest values for all the alpha indices. Chao1 index was significantly lower in the Ma-MS2 group compared with the groups receiving MS1 (Ma-MS1, A-MS1) (*p* < 0.05) and tended to be lower in the CON vs. A-MS1 group (*p* = 0.07) and in the CON vs. MS1 groups (*p* = 0.06). Also, at d21, the Shannon index was significantly lower in the CON group compared with the MS1 groups (*p* < 0.05) and tended to be lower in the CON compared to A-MS1 and Ma-MS1 groups (*p* = 0.08 and *p* = 0.09, respectively) and in the Ma-MS2 vs. MS1 (*p* = 0.05). InvSimpon index was significantly lower in the CON vs MS1 (*p* = 0.01), in the CON vs. A-MS1 (*p* = 0.01) and in the Ma-MS2 vs. MS1 groups (*p* = 0.01) and tended to be lower in the CON vs. Ma-MS1 group (*p* = 0.06) and in the CON vs. milk supplemented groups (A-MS1, Ma-MS1, Ma-MS2) (*p* = 0.07). Alpha diversity indices values according to the group at d14 and d21 are reported in Figure 2.

**Figure 3:**
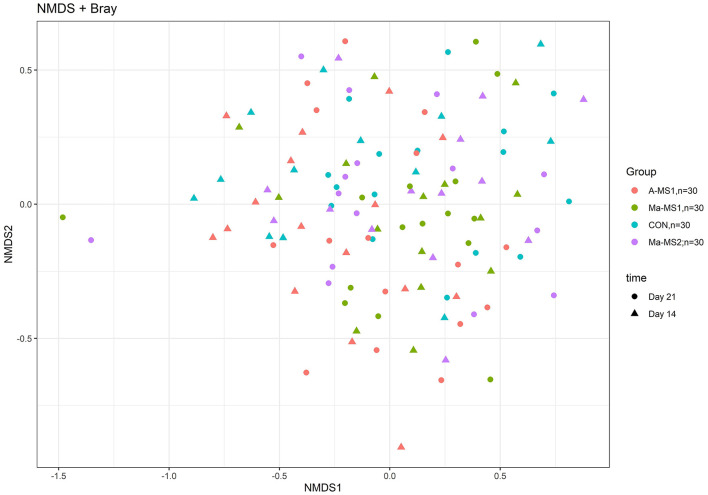
Non-metric multi-dimensional scaling (NMDS) on Bray-Curtis distances at ASVs level. CON, control group without milk supplementation; A-MS1, Milk replacer supplementation 1, provided automatically; Ma-MS1, Milk replacer supplementation 1, provided manually twice a day; Ma-MS2, Milk replacer supplementation 2, provided manually twice a day.

For the beta diversity, Figure 3 shows the non-metric multidimensional scaling (NMDS) plot using the Bray-Curtis distance matrix; the samples belonging to the different groups and time points were partially overlapping and no defined cluster can be observed. The group significantly affected the Beta diversity at d14 (*p* = 0.03; R2 = 0.07) and tended to influence it at d21 (*p* = 0.06; R2 = 0.06).

**Figure 4:**
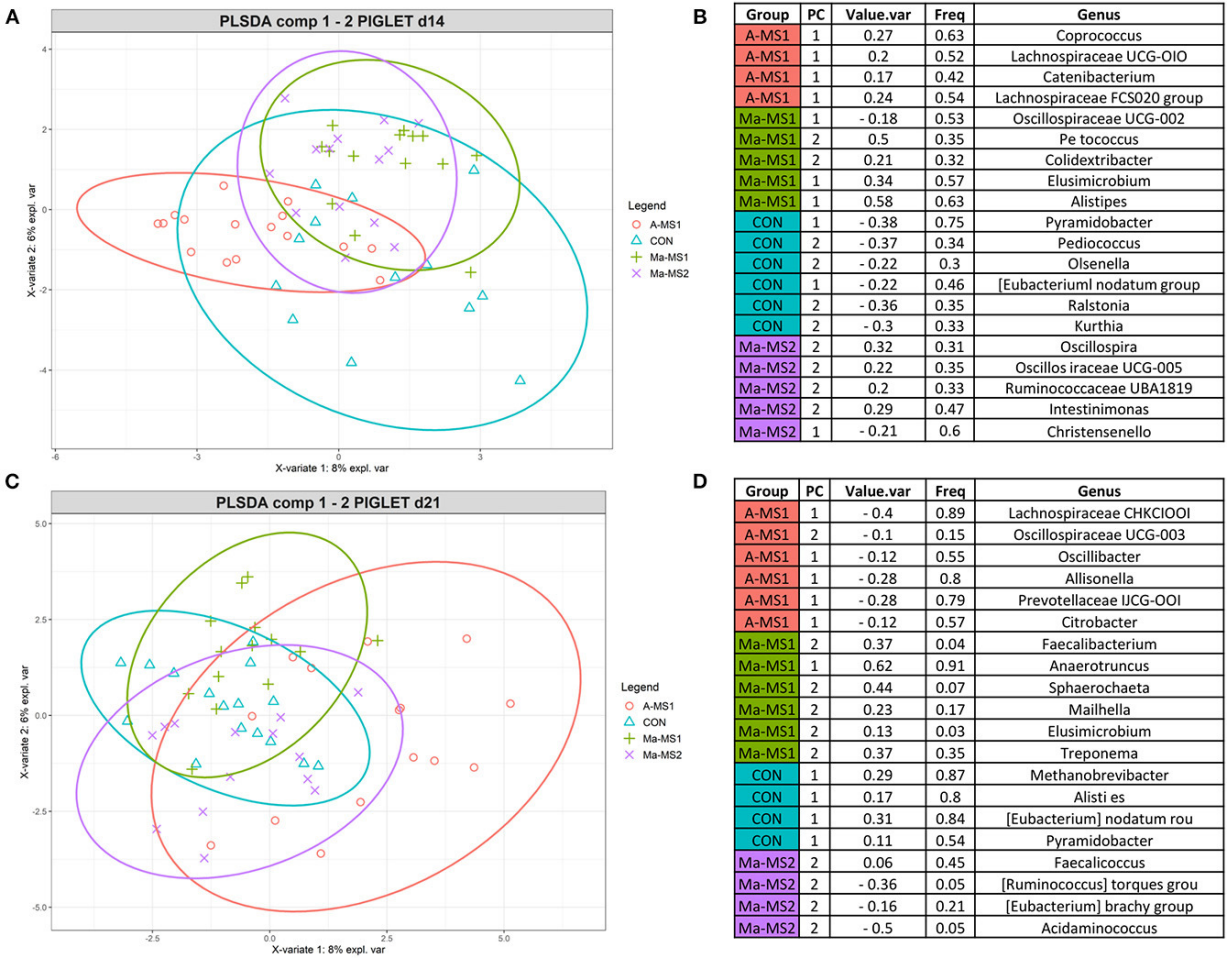
Results of the PLS-DA analysis on fecal microbiota of piglets at d14 **(A, C)** Individual score plots of the samples along the first two components at d14 **(A)** and d21 **(C)**. **(B, D)** Table reporting the most discriminant genera per group at d14 **(B)** and d21 **(D)**; Value.var expresses the variance explained by the single genera; Freq, express the frequencies by which the genera were chosen among the 100 repetitions of the cross validation; PC stands for the principal component that discriminate the genera; Group express the group for which the genera were discriminant. CON, control group without milk supplementation; A-MS1, MS1, provided automatically; Ma-MS1, MS1, provided manually twice a day; Ma-MS2, MS2, provided manually twice a day.

To identify the discriminant taxa that belonged to the specific groups, the multivariate supervised approach Partial Least Squares Discriminant Analysis (PLS-DA) was performed on the data for the two time points at the genus level (Figure 4). At d14, the group A-MS1 was discriminated by bacteria belonging to the genera *Coprococcus*, Lachnospiraceae UCG-010, *Catenibacterium* and Lachnospiraceae FCS020 group; the group Ma-MS1 by bacteria belonging to the genera Oscillospiraceae UCG-002, *Peptococcus, Colidextribacter, Elusimicrobium* and *Alistipes*; the group CON was discriminated by bacteria belonging to the genera Pyramidobacter, *Pediococcus, Olsenella, Eubacterium nodatum* group, *Ralstonia* and *Kurthia* and the group Ma-MS2 by bacteria belonging to the genera *Oscillospira*, Oscillospiraceae UCG-005, Ruminococcaceae UBA1819, *Intestinimonas* and *Christensenella* (Figure 4B). At d21, A-MS1 group was discriminated by bacteria belonging to the genera Lachnospiraceae CHKCI001, Oscillospiraceae UCG-003, *Oscillibacter, Allisonella*, Prevotellaceae UCG-001, *Citrobacter*; the Ma-MS1 group by bacteria belonging to the genera *Faecalibacterium, Anaerotruncus, Sphaerochaeta, Mailhella, Elusimicrobium* and *Treponema*; the group CON was discriminated by bacteria belonging to the genera *Methanobrevibacter, Alistipes, Eubacterium nodatum* group and the group Ma-MS2 by bacteria belonging to the genera *Pyramidobacter, Faecalicoccus, Rumunicoccus torques* group, *Eubacterium brachy* group and *Acidaminococcus* (Figure 4D).

## 4. Discussion

This study compared the effect of two different MS formulations and for the first time compared two ways of administering MS in order to provide indications on the effectiveness of the continuous availability of MS freshly prepared and distributed with an automatic pipeline compared to the classical way of MS supplementation based on the distribution of the MS twice a day in a trough, all groups were suckling the sow and were provided with creep feed.

In the present study, the pre-weaning mortality in the period from d0 (day 5 after birth) to d14 and in the overall pre-weaning period was significantly reduced in the A-MS1 and Ma-MS1 groups, as compared to CON and Ma-MS2 groups (which were similar in their mortality rate). In addition, the continuous access to the same formula was more effective in reducing the mortality compared to the manual supplementation. This result highlights that MS composition and its continuous availability could reduce piglet's mortality. The availability of MS released on demand could have been more attractive for the lighter piglets, potentially reducing the risk of starvation. In agreement with the present study, Novotni-Dankó et al. (27) reported a reduction in pre-weaning mortality using milk supplement from d10 after birth until the end of the suckling period, while an earlier supplementation tended to improve [d3–21d of age, (28)] or did not improve piglet's survival during the whole suckling period (29). Different responses to MS, in terms of piglets mortality, may be related to the litter size, which was bigger in our study compared to the latter studies (13 vs. 10 piglets per sow), and to the different compositions of the MS used in the studies (30). Moreover, as our trial was performed during summer, and the sows were exposed to temperature higher than 26°C, the milk production could have been impaired due to limited feed intake that is observed during heat stress (7), leading to a potential higher use of the MS from the piglets. Futhermore, Kobek-Kjeldager et al. (10) showed how MS can reduce mortality starting from the day 5 after birth, as it was not effective to reduce mortality in first days, where the mortality is usually higher, as piglets may not have learned to use MS yet, which may limit the early effects of the MS. Alongside the way of administration, MS composition appears to be an important factor in determining its effectiveness; indeed, MS2 was not so effective in reducing mortality compared with MS1. However, the MS compositions were quite similar, as the same type of probiotics were used and also energetic density, vitamins, and minerals composition are similar, the major difference of the two formulas are on the type of sweeteners used, since the MS2 contained artificial sweeteners compared to sweetened whey in MS1. Unfortunately, since the milk intake was not measured, we can only assume that the palatability of the formula can explain these differences.

Nevertheless, growth parameters showed no significant differences among groups, in contrast with previous studies that reported increased BW (27) and ADG at weaning in the whole litter (27–29, 31) as well as in the low BW piglets (11) in piglets receiving MS as compared to unsupplemented ones. These contrasting results might be attributed to differences in the duration of milk supplementation among studies. In the cited studies, the MS were available until weaning, while in the present study the MS was interrupted 1 week before weaning. Furthermore, the absence of difference in the growth performance among the groups observed in the present study should be evaluated considering the higher survivability of the pigs in the A-MS1 group. Indeed, the higher death percentage observed in the CON, Ma-MS2 and Ma-MS1 groups compared to A-MS1, could have determined an advantage in terms of nutrient availability and, in turn, body condition for survived piglets. Although data regarding the effect of MS in the post-weaning phase are very scarce, Wolter et al. (28) and Azain et al. (29) reported no differences in the growth performance from weaning to slaughter in piglets reared using MS during the suckling phase.

Behavioral analysis showed that at d4 litters fed the MS had a higher frequency of negative social behavior and pen exploration. The former can be considered a sign of distress in piglets as the response to a barren environment or stressful rearing condition, while the latter may come from various needs including searching for food, acquiring information on the surrounding (curiosity) or escape from boredom (32). Although not directly comparable, a previous study has reported increased aggressive behavior in piglets raised artificially with MS compared with piglets reared without. The authors associated this results to small space allowance in the tested artificial rearing system (33). Since negative social behaviors were recorded just at d4 and not at the other timepoints, we can indirectly assume that in the first days after the automatic system activation, there was a higher competition for the access to the drinking cup, confirming the palatability of the tested MS. These negative behaviors were not observed later, as a stable hierarchy was eventually formed for the access to the milk cup. However, we did not directly measure competition for the cup and this aspect should be clarified in further studies. Moreover, LBs showed that later in the nursery phase (d10), Ma-MS2 piglets had higher teat competition and the lowest percentage of piglets not eating during the nursing events, later on this group had the highest percentage of piglets not eating compared to the others (d18). This suggests that at day 10 in the Ma-MS2 group most of the piglets were involved in fights for the access to the teat and a stable hierarchy was not yet formed while later when teat competition was stable between groups, and a stable hierarchy was presumably formed, most the piglets of the Ma-MS2 group where not eating. Considering these parameters together, and given the higher mortality observed in the Ma-MS2 group, it can be hypothesized that Ma-MS2 piglets showed a signal of worse welfare. However, this behavior was limited to a certain day, and no effects were seen in the lesion score or performance, so the negative effect of Ma-MS2 on piglets welfare cannot be concluded from this trial.

In this study, the MS affected the fecal microbial ecosystem of piglets both during the period in which piglets were fed the MS (d14) and 1 week after the interruption of MS administration (d21). Few studies have investigated the effect of MS on the microbial profile of piglets. Previous studies have shown a significant effect of sow milk or MS in influencing the microbial profile (13, 34). In the present study, both the MSs increased the microbial diversity indices compared with the CON group at d14, as also observed by Jin et al. (13), while at d 21 this effect was maintained only for the groups receiving MS1 indicating a significant effect of this milk formula in influencing the gut microbial profile. From an ecological perspective, an increase of alpha diversity indices has been associated with a more mature and stable microbial profile and the increase of bacterial species in the community would favor the functional redundancy contrasting stressful events that may lead to dysbiosis conditions (35). The supplementation of MS during suckling led also to differentiate the microbial structure and composition, as highlighted by the results for the beta diversity indicating that the similarity among samples in the same groups is higher than the similarity among samples in the different groups. The differences in the alpha and beta indices, taxonomy relative abundance and the sPLS-DA indicated that the differences among samples in the groups were more evident on d14 compared to d21. This can be due to the intake of the solid feed, supplied from the d15 that can flatten the gut microbiota diversity among groups. At d14, the groups that received the MS1 were characterized by microbial genera recognized as producers of short-chain fatty acids (SCFAs), namely those belonging to the Lachnospiraceae (A-MS1 group) (36), Oscillospiraceae (Ma-MS1 group) families, *Alistipes* (Ma-MS1 group) (37) and *Coprococcus* (A-MS1 group) (38) genera. We can assume that SCFAs producing bacteria might have contributed to a favorable gut microbial environment in the piglets receiving the MS1 formula. Nevertheless, important SCFAs producer bacteria, like Ruminocacceae (36), were also recovered in the Ma-MS2 group (d14), however, both Ma-MS2 and CON were also characterized by the presence of bacteria like *Eubacterim brachy* group (d21) and *Eubacterim nodatum* (both d14 and d21) group, that are considered opportunistic pathogens in humans (39). It is known that early commensal colonization of piglets' intestinal microbiota can influence piglets' robustness in experimental conditions, with a beneficial effect on the development of the intestinal and systemic immune system and nutrients absorption, resulting in better growth performance and health (40). In line with these assumptions, the higher presence of potentially beneficial bacteria observed in A-MS1 and Ma-MS1 groups, and the higher presence of opportunistic pathogens in the Ma-MS2 and CON groups suggest that MS1 could have favored the establishment of a different microbial profile and may have increased the intestinal microbial eubiosis (41). Nonetheless, 16s amplicon sequencing have limitations that need to be considered when interpreting the results, indeed the use of more sensitive technologies like whole genome shotgun sequencing can give a clearer picture of the changes that may occurred (42). In addition, SCFAs and diarrhea incidence was not directly measured in our study and need to be carefully considered in further studies.

Moreover, as the effect of the MS is likely dependent on the intake level, and some of the sampled piglets may not have been consumers of MS, the effect observed may have been underestimated in this study (9). In addition, it may be interesting to link the amount of MS intake to the observed effects, therefore, this requires careful consideration in future research.

## 5. Conclusion

Overall, the results obtained in this study suggest that MS can contribute to the reduction of piglet's pre-weaning mortality and that its contribution is affected by both milk composition and the way of administration/duration of MS availability during the day. In addition, MS availability did not affect pre- and post-weaning performance and can increase some negative social behaviors in the first days of administration. Finally, the MS affected the overall fecal microbiota profile and the MS1 (A-MS1 and Ma-MS1) favored the presence of SCFAs producing bacteria.

## Data availability statement

The original contributions presented in the study are publicly available. This data can be found at: https://www.ncbi.nlm.nih.gov/bioproject/PRJNA794033.

## Ethics statement

The animal study was reviewed and approved by Ethical Committee of the University of Bologna. Written informed consent was obtained from the owners for the participation of their animals in this study.

## Author contributions

PT and PB designed the experiment, conceptualized the paper, provided insights to the entire manuscript, and contributed to the writing. FC, DL, RR, and CN performed the experiment and collected samples. DL, FC, and CN analyzed samples and analyzed the data. FC and DL conceptualized the paper, compiled all the information, and prepared the manuscript. All authors read and approved the final manuscript.
