# Supplementary material for: Effect of two milk supplements and two ways of administration on growth performance, welfare and fecal microbial ecology of suckling piglets
Source: Front Vet Sci. 2023 Feb 27;10:1050414. doi: 10.3389/fvets.2023.1050414 (PMC10008956; doi:10.3389/fvets.2023.1050414)
Supplement: Supplementary file 1 [file Data_Sheet_1.docx]

Supplementary Material

**Supplementary Figure 1.** Rarefaction curves of samples resulted by sequencing of V3–V4 regions with MiSeq platform (Illumina Inc., San Diego, Ca, USA)


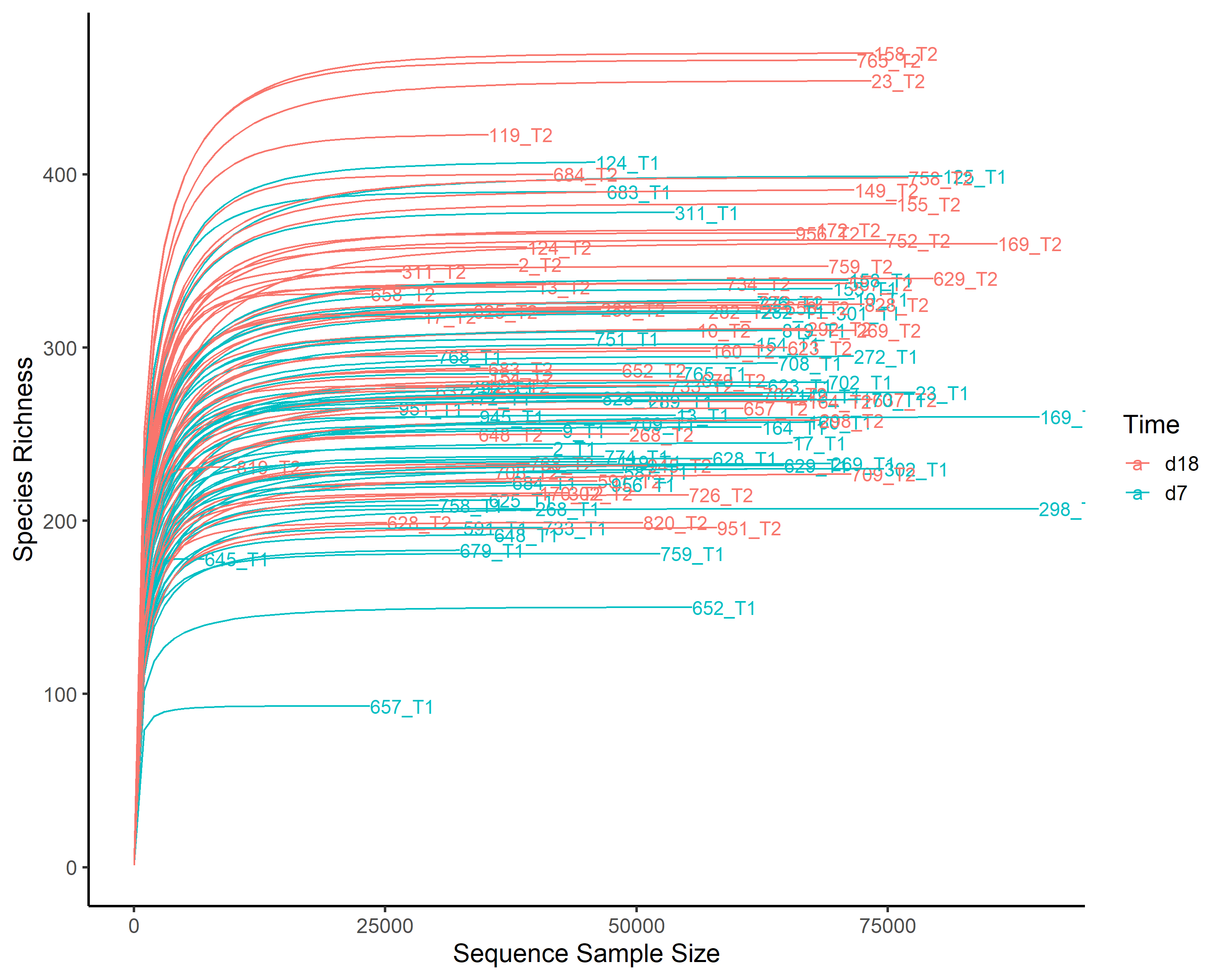


**Supplementary Table 1.** Effect of milk supplementation on behavioural indicators of the litters**.** Data are reported as % of piglets involved in that behavior in the litter,

| Item | Mean^1^ | | | | SEM | *p*-value^2^ | | |
| --- | --- | --- | --- | --- | --- | --- | --- | --- |
|  | CON, n=5 | A-MS1, n=5 | Ma-MS1, n=5 | Ma-MS2, n=5 |  | Group | Litter size | CON vs MS |
| Behaviour day 4 (%) | | | | | | | | |
| Inactive behaviour | 84.50 | 64.50 | 75.90 | 49.80 | 8.59 | 0.06 | 0.40 | **0.06** |
| Negative social behaviour | 1.22 | 4.08 | 4.35 | 7.76 | 1.54 | 0.06 | 0.39 | **0.03** |
| Positive social behaviour | 1.56 | 1.05 | 0.90 | 4.64 | 1.38 | 0.22 | 0.32 | 0.63 |
| Pen exploration | 3.28^a^ | 18.4^ab^ | 7.45^ab^ | 19.85^b^ | 3.91 | 0.02 | 0.60 | **0.02** |
| Environmental enrichment exploration | 0.00 | 0.39 | 0.00 | 2.60 | 0.89 | 0.16 | 0.78 | 0.48 |
| Other active behaviour | 9.44 | 11.59 | 11.40 | 15.33 | 5.73 | 0.91 | 0.38 | 0.57 |
| Behaviour day 10 (%) | | | | | | | | |
| Inactive behaviour | 44.10 | 37.30 | 42.40 | 53.10 | 7.43 | 0.52 | 0.052 | 0.71 |
| Negative social behaviour | 3.84 | 9.70 | 4.39 | 4.35 | 2.07 | 0.18 | 0.80 | 0.42 |
| Positive social behaviour | 3.14 | 4.00 | 7.18 | 3.25 | 1.83 | 0.39 | 0.10 | 0.34 |
| Pen exploration | 33.90 | 34.80 | 20.00 | 29.10 | 5.06 | 0.18 | 0.11 | 0.56 |
| Environmental enrichment exploration | 8.01 | 5.39 | 8.06 | 3.04 | 1.57 | 0.12 | 0.07 | 0.08 |
| Other active behaviour | 7.00 | 8.75 | 18.03 | 7.14 | 4.07 | 0.22 | 0.94 | 0.23 |
| Behaviour day 18 (after milk suspension) (%) | | | | | | |  |  |
| Inactive behaviour | 29.60 | 34.20 | 48.50 | 54.40 | 9.70 | 0.25 | 0.91 | 0.13 |
| Negative social behaviour | 7.25 | 3.53 | 5.91 | 3.56 | 2.07 | 0.51 | 0.90 | 0.31 |
| Positive social behaviour | 8.14 | 6.74 | 5.30 | 3.82 | 3.24 | 0.80 | 0.46 | 0.57 |
| Pen exploration | 30.40 | 30.50 | 28.20 | 21.5 | 5.53 | 0.63 | 0.06 | 0.45 |
| Environmental enrichment exploration | 6.08 | 2.98 | 3.72 | 5.90 | 2.09 | 0.67 | 0.05 | 0.43 |
| Other active behaviour | 18.47 | 22.03 | 8.34 | 10.83 | 5.19 | 0.23 | 0.14 | 0.38 |

^a,b,c^ = values with different superscript differs (*p* < 0.05).

^1^CON = control group without milk supplementation; A-MS1 = MS1, provided automatically; Ma-MS1 = MS1, provided manually twice a day; Ma-MS2 = MS2, provided manually twice a day. Values are least estimate means of the values.

^2^CON vs MS = CON vs litters receiving MS (A-MS1, Ma-MS1 and Ma-MS2); no significant effect for Automatic vs Manual Supplementation (A-MS1 vs Ma-MS1+Ma-MS2) and for A-MS1 vs A-MS2

Supplementary Table 2. Effect of milk supplementation on behaviour during the suckling event. Data are reported as % of piglets involved in that behavior in the litter,

| Item | Mean^1^ | | | | | | SEM | *p*-value^2^ | | | | |
| --- | --- | --- | --- | --- | --- | --- | --- | --- | --- | --- | --- | --- |
|  | CON, n=5 | A-MS1, n=5 | | Ma-MS1, n=5 | | Ma-MS2, n=5 |  | Group | Litter size | Teat exposition score | Working  nipples (n) |  |
| *Behaviour d 4 (%)* | | | | | | | | | | | |  |
| Teat competition | 7.92 | 2.94 | 5.06 | | 5.82 | | 1.53 | 0.21 | 0.49 | 0.43 | < 0.01 |  |
| Not eating | 9.04 | 11.92 | 13.64 | | 10.67 | | 2.31 | 0.61 | 0.42 | 0.42 | 0.09 |  |
| Supplementary milk | - | 2.96 | 1.58 | | 2.61 | | 1.50 | 0.44 | 0.31 | 0.88 | 0.49 |  |
| *Behaviour d 10 (%)* | | | | | | | | | | | |  |
| Teat competition | 1.44^ab^ | 3.34^ab^ | 0.51^a^ | | 6.26^b^ | | 1.26 | 0.03 | 0.16 | 0.87 | 0.98 |  |
| Not eating | 8.97 | 11.85 | 7.27 | | 2.57 | | 2.00 | 0.07 | 0.47 | 0.91 | 0.29 |  |
| Supplementary milk | - | 0.09 | 0.00 | | 0.41 | | 0.27 | 0.66 | 0.96 | 0.61 | 0.25 |  |
| *Behaviour d 18 (after milk suspension) (%)* | | | | | | | | | | | |  |
| Teat competition | 1.49 | 1.46 | 3.96 | | 4.92 | | 1.58 | 0.34 | 0.51 | 0.74 | 0.53 |  |
| Not eating | 8.58^ab^ | 3.12^a^ | 5.99^a^ | | 12.36^b^ | | 1.71 | < 0.01 | 0.004 | 0.14 | < 0.01 |  |

^a,b,c^ = values with different superscript differs (*p* < 0.05).

^1^CON = control group without milk supplementation; A-MS1 = MS1, provided automatically; Ma-MS1 = MS1, provided manually twice a day; Ma-MS2 = MS2, provided manually twice a day

^2^ No significant effect for CON vs MS = Control group vs litters receiving MS (A-MS1, Ma-MS1 and Ma-MS2) and for Automatic vs Manual supplementation (A-MS1 vs Ma-MS1+Ma-MS2).

**Supplementary Table 3.** Effect of nursery milk supplementation on lesion score index considering the different body weight

| Item | Mean (score)^1^ | | | | SEM | *p* – value^2^ | | | | | |
| --- | --- | --- | --- | --- | --- | --- | --- | --- | --- | --- | --- |
|  | CON, n=5 | A-MS1, n=5 | Ma-MS1, n=5 | Ma-MS2, n=5 |  | Group | BW category^3^ | CON vs Milk | Automatic vs Manual | Ma-MS1  vs  Ma-MS2 | Ma-MS1  vs  A-MS1 |
| Lesions day 4 | | | | | | | | | | | |
| Ear | 0.10 | 0.20 | 0.00 | 0.0 | 0.07 | 0.18 | 0.07 | 0.86 | 0.03 | 0.87 | 0.07 |
| Front | 0.50 | 0.60 | 0.30 | 0.7 | 0.15 | 0.42 | 0.83 | 0.93 | 0.61 | 0.12 | 0.24 |
| Middle | 0.00 | 0.20 | 0.00 | 0.0 | 0.07 | 0.21 | 0.13 | 0.52 | 0.05 | 0.78 | 0.10 |
| Hind quarter | 0.00 | 0.00 | 0.00 | 0.0 | 0.01 | 0.52 | 0.29 | 0.37 | 0.44 | 0.35 | 0.26 |
| Tail | 0.00 | 0.00 | 0.00 | 0.0 | 0.02 | 0.46 | 0.11 | 0.75 | 0.89 | 0.12 | 0.39 |
| Lesions day 10 | | | | | | | | | | |  |
| Ear | 0.00 | 0.20 | 0.10 | 0.00 | 0.05 | 0.15 | 0.57 | 0.23 | 0.17 | 0.13 | 0.62 |
| Front | 0.30 | 0.40 | 0.30 | 0.30 | 0.14 | 1.00 | 0.04 | 0.96 | 0.85 | 0.91 | 0.83 |
| Middle | 0.00 | 0.10 | 0.10 | 0.00 | 0.04 | 0.17 | 0.29 | 0.11 | 0.24 | 0.22 | 0.65 |
| Hind quarter | 0.00 | 0.10 | 0.10 | 0.00 | 0.02 | 0.03 | 0.52 | 0.09 | 0.49 | 0.02 | 0.61 |
| Tail | 0.00 | 0.20 | 0.10 | 0.10 | 0.07 | 0.67 | 0.62 | 0.31 | 0.52 | 0.79 | 0.49 |
| Lesions day 18 | | | | | | | | | | |  |
| Ear | 0.10 | 0.10 | 0.20 | 0.10 | 0.06 | 0.88 | 0.10 | 0.70 | 0.98 | 0.48 | 0.71 |
| Front | 0.30 | 0.30 | 0.30 | 0.40 | 0.12 | 0.70 | 0.06 | 0.72 | 0.43 | 0.44 | 0.74 |
| Middle | 0.00 | 0.20 | 0.10 | 0.00 | 0.08 | 0.41 | 0.04 | 0.47 | 0.18 | 0.44 | 0.40 |
| Hind quarter | 0.00 | 0.10 | 0.10 | 0.10 | 0.04 | 0.93 | 0.57 | 0.53 | 0.92 | 0.94 | 0.96 |
| Tail | 0.00 | 0.10 | 0.10 | 0.10 | 0.03 | 0.74 | 0.24 | 0.40 | 0.65 | 0.59 | 0.89 |

^1^CON = control group without milk supplementation; A-MS1 = MS1, provided automatically; Ma-MS1 = MS1, provided manually twice a day; Ma-MS2 = MS2, provided manually twice a day. Values are least estimate means of the values.

^2^CON vs MILK = Control group vs litters receiving milk supplementation (thus A-MS1, Ma-MS1 and Ma-MS2); Automatic vs Manual Supplementation (A-MS1 vs Ma-MS1+Ma-MS2).

^3^ Each piglet was assigned to a class of weight (BW category = heavy, middle, light) according to the quartile distribution of the litter.

**Supplementary Table 4.** List of number of reads (Depth), Observed ASVs, Chao1, Shannon and InvSimpson indices in piglet

| Piglet | Time | Group^1^ | Depth | Observed ASV | Chao1 | Shannon | InvSimpson |
| --- | --- | --- | --- | --- | --- | --- | --- |
| 768 | Day 21 | A-MS1 | 39364 | 233 | 233 | 3.7 | 11.64 |
| 768 | Day 14 | A-MS1 | 30223 | 295 | 295 | 4.53 | 32.5 |
| 625 | Day 21 | A-MS1 | 33773 | 321 | 321 | 4.6 | 45.67 |
| 17 | Day 21 | A-MS1 | 28703 | 318 | 318 | 4.49 | 35.87 |
| 124 | Day 14 | A-MS1 | 45915 | 407 | 407.25 | 4.8 | 56.91 |
| 625 | Day 14 | A-MS1 | 35297 | 212 | 212.5 | 3.7 | 15.13 |
| 628 | Day 21 | A-MS1 | 25175 | 199 | 199.5 | 4.3 | 38.4 |
| 124 | Day 21 | A-MS1 | 39114 | 358 | 358 | 4.77 | 56.06 |
| 951 | Day 14 | A-MS1 | 26334 | 265 | 265 | 4.53 | 46.96 |
| 119 | Day 21 | A-MS1 | 35262 | 423 | 423.75 | 4.94 | 57.83 |
| 774 | Day 14 | A-MS1 | 46812 | 237 | 237 | 4.1 | 29.53 |
| 945 | Day 14 | A-MS1 | 34347 | 260 | 260 | 4.26 | 28.14 |
| 125 | Day 14 | A-MS1 | 80417 | 399 | 399 | 4.79 | 56.46 |
| 13 | Day 14 | A-MS1 | 53902 | 261 | 261 | 3.91 | 14.97 |
| 17 | Day 14 | A-MS1 | 65526 | 245 | 245 | 3.08 | 4.53 |
| 23 | Day 21 | A-MS1 | 73347 | 454 | 454 | 5.05 | 79.83 |
| 623 | Day 21 | A-MS1 | 65024 | 300 | 300 | 4.27 | 23.94 |
| 119 | Day 14 | A-MS1 | 48054 | 234 | 234 | 4.37 | 40.94 |
| 23 | Day 14 | A-MS1 | 77744 | 274 | 274 | 4.22 | 32.01 |
| 629 | Day 14 | A-MS1 | 64685 | 232 | 232 | 3.15 | 7.28 |
| 629 | Day 21 | A-MS1 | 79543 | 340 | 340.33 | 4.54 | 49.87 |
| 951 | Day 21 | A-MS1 | 58012 | 196 | 196 | 3.71 | 19.64 |
| 956 | Day 21 | A-MS1 | 65790 | 366 | 366 | 4.58 | 33.45 |
| 623 | Day 14 | A-MS1 | 63036 | 278 | 278.33 | 4.17 | 16.57 |
| 765 | Day 14 | A-MS1 | 54543 | 285 | 285 | 4.28 | 26.69 |
| 765 | Day 21 | A-MS1 | 71904 | 466 | 466 | 5.05 | 69.89 |
| 956 | Day 14 | A-MS1 | 47427 | 221 | 222 | 3.56 | 8.44 |
| 628 | Day 14 | A-MS1 | 57578 | 236 | 236 | 3.91 | 22.48 |
| 945 | Day 21 | A-MS1 | 51057 | 232 | 232 | 3.59 | 11.91 |
| 125 | Day 21 | A-MS1 | 34960 | 278 | 278 | 4.73 | 64.55 |
| 13 | Day 21 | A-MS1 | 40039 | 335 | 335 | 4.85 | 63.76 |
| 828 | Day 14 | Ma-MS1 | 46564 | 271 | 271 | 4.29 | 30.21 |
| 311 | Day 21 | Ma-MS1 | 26666 | 344 | 344.1 | 4.83 | 63.67 |
| 758 | Day 14 | Ma-MS1 | 30299 | 209 | 209 | 4.03 | 24.46 |
| 154 | Day 21 | Ma-MS1 | 35350 | 283 | 283 | 4.08 | 22.27 |
| 172 | Day 14 | Ma-MS1 | 33056 | 271 | 271 | 4.57 | 48.94 |
| 154 | Day 14 | Ma-MS1 | 61824 | 302 | 302 | 4.09 | 22.51 |
| 155 | Day 14 | Ma-MS1 | 69503 | 334 | 334.5 | 4.66 | 50.54 |
| 172 | Day 21 | Ma-MS1 | 67869 | 368 | 368 | 4.3 | 21.53 |
| 301 | Day 14 | Ma-MS1 | 69820 | 320 | 320 | 4.46 | 38.42 |
| 302 | Day 14 | Ma-MS1 | 74630 | 230 | 230 | 4.09 | 30.93 |
| 311 | Day 14 | Ma-MS1 | 53779 | 378 | 378 | 4.75 | 52.32 |
| 149 | Day 14 | Ma-MS1 | 65891 | 272 | 272 | 4.44 | 42.15 |
| 158 | Day 21 | Ma-MS1 | 73586 | 470 | 470 | 5.12 | 78.88 |
| 160 | Day 14 | Ma-MS1 | 67016 | 257 | 257 | 4.07 | 26.96 |
| 752 | Day 21 | Ma-MS1 | 74844 | 362 | 362 | 4.59 | 35.92 |
| 819 | Day 14 | Ma-MS1 | 64480 | 310 | 310 | 4.57 | 45.91 |
| 751 | Day 14 | Ma-MS1 | 45835 | 305 | 305 | 4.42 | 35.97 |
| 758 | Day 21 | Ma-MS1 | 77064 | 398 | 398 | 4.68 | 42.23 |
| 759 | Day 14 | Ma-MS1 | 52355 | 181 | 181 | 4.15 | 33.17 |
| 759 | Day 21 | Ma-MS1 | 69115 | 347 | 347 | 4.42 | 34.74 |
| 819 | Day 21 | Ma-MS1 | 10182 | 231 | 231 | 4.59 | 50.07 |
| 828 | Day 21 | Ma-MS1 | 72667 | 325 | 325.33 | 4.41 | 31.38 |
| 155 | Day 21 | Ma-MS1 | 75848 | 383 | 383 | 4.81 | 54.77 |
| 158 | Day 14 | Ma-MS1 | 71104 | 339 | 339 | 4.38 | 30.45 |
| 149 | Day 21 | Ma-MS1 | 71656 | 391 | 391.33 | 4.81 | 54.77 |
| 820 | Day 21 | Ma-MS1 | 50652 | 199 | 199 | 3.62 | 15.87 |
| 160 | Day 21 | Ma-MS1 | 57343 | 298 | 298 | 4.23 | 22.12 |
| 302 | Day 21 | Ma-MS1 | 43238 | 216 | 216 | 4.21 | 35.19 |
| 683 | Day 21 | CON | 35251 | 288 | 288.17 | 4.34 | 27.13 |
| 637 | Day 21 | CON | 30038 | 275 | 275 | 4.43 | 45.23 |
| 648 | Day 21 | CON | 34267 | 250 | 252 | 4.28 | 34.93 |
| 591 | Day 14 | CON | 32756 | 196 | 196 | 3.66 | 14.58 |
| 679 | Day 14 | CON | 32399 | 183 | 183 | 3.95 | 28.89 |
| 169 | Day 21 | CON | 85920 | 360 | 360 | 4.22 | 27 |
| 587 | Day 14 | CON | 48693 | 228 | 228 | 3.63 | 9.12 |
| 652 | Day 14 | CON | 55520 | 150 | 150 | 3.67 | 17.38 |
| 679 | Day 21 | CON | 56481 | 281 | 281 | 4.26 | 24.01 |
| 683 | Day 14 | CON | 47038 | 390 | 390 | 5.04 | 87.86 |
| 637 | Day 14 | CON | 73472 | 270 | 270 | 4.4 | 41.54 |
| 645 | Day 14 | CON | 7038 | 178 | 178 | 4.42 | 45.88 |
| 164 | Day 14 | CON | 62455 | 254 | 254 | 4.04 | 24.98 |
| 298 | Day 14 | CON | 90017 | 207 | 207 | 3.05 | 7 |
| 298 | Day 21 | CON | 68207 | 258 | 258 | 3.5 | 9.09 |
| 587 | Day 21 | CON | 71071 | 337 | 337 | 4.44 | 34.18 |
| 170 | Day 14 | CON | 72307 | 270 | 270 | 3.81 | 20.7 |
| 591 | Day 21 | CON | 46119 | 223 | 223 | 3.58 | 11.73 |
| 658 | Day 14 | CON | 23569 | 331 | 331 | 4.27 | 17.46 |
| 657 | Day 21 | CON | 60639 | 265 | 265 | 4.18 | 20.14 |
| 657 | Day 14 | CON | 23458 | 93 | 93 | 3.17 | 8.31 |
| 658 | Day 21 | CON | 64812 | 323 | 323 | 4.6 | 48.79 |
| 684 | Day 14 | CON | 37597 | 222 | 225 | 4.2 | 32.42 |
| 684 | Day 21 | CON | 41710 | 400 | 400 | 4.68 | 28.23 |
| 170 | Day 21 | CON | 40284 | 216 | 217 | 3.85 | 20.4 |
| 164 | Day 21 | CON | 66983 | 269 | 269 | 4.26 | 39.22 |
| 648 | Day 14 | CON | 35833 | 192 | 192.33 | 3.7 | 15.25 |
| 169 | Day 14 | CON | 90093 | 260 | 260 | 3.81 | 21.09 |
| 652 | Day 21 | CON | 48558 | 287 | 287 | 4.35 | 24.87 |
| 708 | Day 21 | Ma-MS2 | 35886 | 228 | 228 | 4.2 | 32.86 |
| 268 | Day 14 | Ma-MS2 | 39919 | 207 | 207 | 3.5 | 8.62 |
| 292 | Day 14 | Ma-MS2 | 33384 | 277 | 277 | 4.48 | 45.45 |
| 9 | Day 14 | Ma-MS2 | 42602 | 252 | 252 | 3.85 | 13.8 |
| 10 | Day 14 | Ma-MS2 | 71697 | 328 | 329 | 4.65 | 49.7 |
| 10 | Day 21 | Ma-MS2 | 56023 | 310 | 311.5 | 4.52 | 36.7 |
| 269 | Day 14 | Ma-MS2 | 69355 | 233 | 233 | 4.15 | 35.31 |
| 269 | Day 21 | Ma-MS2 | 71921 | 310 | 310 | 4.1 | 22.62 |
| 702 | Day 14 | Ma-MS2 | 69039 | 280 | 280 | 4.48 | 44.24 |
| 708 | Day 14 | Ma-MS2 | 64043 | 291 | 291 | 4.29 | 22.84 |
| 726 | Day 14 | Ma-MS2 | 61959 | 326 | 326 | 4.39 | 34.98 |
| 726 | Day 21 | Ma-MS2 | 55210 | 215 | 215 | 3.7 | 15.04 |
| 292 | Day 21 | Ma-MS2 | 66989 | 311 | 311.33 | 4.26 | 25.82 |
| 272 | Day 14 | Ma-MS2 | 71616 | 295 | 295 | 4.49 | 35.17 |
| 272 | Day 21 | Ma-MS2 | 62197 | 326 | 326 | 4.52 | 45.59 |
| 282 | Day 21 | Ma-MS2 | 57169 | 321 | 322 | 4.44 | 31.34 |
| 289 | Day 21 | Ma-MS2 | 46465 | 322 | 322.33 | 4.61 | 40.83 |
| 2 | Day 14 | Ma-MS2 | 41676 | 242 | 242 | 4.47 | 42.74 |
| 268 | Day 21 | Ma-MS2 | 49263 | 250 | 250 | 3.76 | 11.2 |
| 709 | Day 14 | Ma-MS2 | 49436 | 256 | 256 | 4.31 | 37.7 |
| 734 | Day 21 | Ma-MS2 | 58888 | 337 | 337 | 4.37 | 25.4 |
| 733 | Day 14 | Ma-MS2 | 40667 | 196 | 196 | 3.34 | 6.71 |
| 702 | Day 21 | Ma-MS2 | 62548 | 273 | 273 | 4.09 | 23.21 |
| 709 | Day 21 | Ma-MS2 | 71391 | 227 | 227.75 | 3.82 | 18.69 |
| 282 | Day 14 | Ma-MS2 | 62341 | 321 | 321 | 4.78 | 66.52 |
| 289 | Day 14 | Ma-MS2 | 51165 | 269 | 269 | 3.88 | 15.83 |
| 733 | Day 21 | Ma-MS2 | 53249 | 278 | 278 | 3.93 | 15.94 |
| 2 | Day 21 | Ma-MS2 | 38275 | 348 | 348.33 | 4.83 | 44.96 |

^1^CON = control group without milk supplementation; A-MS1 = MS1, provided automatically; Ma-MS1 = MS1, provided manually twice a day; Ma-MS2 = MS2, provided manually twice a day. Values are least estimate means of the values.
